# The Journey of Gene Therapy in Sickle Cell Disease: How Molecular Advances Meet Clinical Care

**DOI:** 10.3390/cells15100939

**Published:** 2026-05-20

**Authors:** Magalie Tardif, Manon Saby, Stéphanie Forté, Thomas Pincez

**Affiliations:** 1 Division of Pediatric Hematology-Oncology, CHU Sainte-Justine, Montreal, QC H3T 1C5, Canada; magalie.tardif.med@ssss.gouv.qc.ca; 2 Faculty of Medicine, Université de Montréal, Montreal, QC H3T 1C5, Canada; 3 CHU Sainte-Justine Azrieli Research Center, Montreal, QC H3T 1C5, Canada; manon.saby.hsj@ssss.gouv.qc.ca; 4 CRCHUM, Montreal, QC H2X 0C1, Canada

**Keywords:** sickle cell disease, hemoglobinopathies, gene therapy, genome editing, CRISPR–Cas9, fetal hemoglobin, *BCL11A*, hematopoietic stem cells, lentiviral vectors, autologous transplantation, exa-cel, lovo-cel

## Abstract

**Highlights:**

**What are the main findings?**

- Gene therapy for sickle cell disease has progressed from lentiviral gene addition strategies to precise genome editing approaches targeting fetal hemoglobin regulation or the β-globin mutation.
- Clinical translation has demonstrated the feasibility of durable disease modification, but implementation remains constrained by conditioning toxicity, delivery challenges, and complex treatment pathways.

**What are the implications of the main findings?**

- Optimizing gene editing technologies and delivery platforms will be essential to improve safety, durability, and physiological control of therapeutic hemoglobin expression.
- Addressing patient selection, long-term outcomes, and global accessibility is critical for translating gene therapy advances into equitable clinical care.

**Abstract:**

Sickle cell disease (SCD) is a monogenic disorder responsible for recurrent vaso-occlusive crises, progressive organ damage, and shortened life expectancy. For decades, allogeneic hematopoietic stem cell transplantation from a matched sibling donor has been the only established cure, but its reach remains limited by donor availability and transplant-related toxicity. The approval of two autologous gene therapy products in 2023, exagamglogene autotemcel (exa-cel) and lovotibeglogene autotemcel (lovo-cel), marked a turning point for the SCD population and the gene therapy field in general. This review proposes a molecular rationale for fetal hemoglobin reactivation and β-globin gene addition, describes the engineering of lentiviral and CRISPR-based platforms, and highlights the clinical evidence accumulated to date that demonstrated durable disease modification with acceptable short-term toxicity. We then assess the clinical positioning of gene therapy within the broader spectrum of curative options compared to current available treatments and address the financial, ethical and psychosocial barriers that limit access to gene therapy both within high-income countries and globally. Critical research priorities include long-term safety surveillance, comparative effectiveness studies, pediatric trials below 12 years, and validated patient-reported outcome instruments. Base editing, non-genotoxic conditioning, and in vivo delivery represent the most promising avenues to broaden access and reduce treatment burden.

## 1. Introduction

Sickle cell disease (SCD) is the most prevalent monogenic disorder worldwide, with close to 8 million individuals estimated to be affected globally [1]. Despite its high global prevalence and its association with early childhood mortality and substantial morbidity, including early-onset stroke in children and severe, largely preventable disabilities, research in this field has historically received less funding than other diseases that predominantly affect populations of European descent [2,3].

SCD was described in 1910 by the Chicago physician James B. Herrick, who reported the case of Walter Noel, a dental student from Grenada, presenting with severe anemia and recurrent pain. During examination of a peripheral blood smear, Herrick’s intern Ernest E. Irons identified unusual crescent-shaped erythrocytes, establishing the first clinicopathologic description of the disorder [4]. Subsequent work by Pauling and colleagues in 1949, using hemoglobin electrophoresis, demonstrated that SCD is caused by a structurally abnormal hemoglobin variant, later termed hemoglobin S (HbS) [5,6]. This seminal work recognized SCD as the first “molecular disease,” demonstrating that a clinical disorder could arise from a specific molecular alteration, laying the foundations of modern molecular medicine [5,7]. Later studies showed that HbS polymerizes under deoxygenated conditions, leading to red blood cell sickling and vaso-occlusive complications [5,6,7,8,9,10]. The molecular basis of the disorder was further elucidated by Ingram, who demonstrated that sickle hemoglobin results from a single amino acid substitution (Glu6Val) in the β-globin chain [11] secondary to a point mutation in the HBB gene [12].

Multiple genotypic forms of this recessive disease have since been recognized, with a declination of clinical phenotypes. The heterozygous combination of HbS and hemoglobin C (HbC), called HbSC disease, produces typical manifestations of SCD, although they often present later in life with a more insidious symptomatology [7,10]. HbC results from a different point mutation in the β-globin gene (Glu6Lys) and tends to form intracellular crystals in oxygenated erythrocytes, promoting red blood cell dehydration and an increase in intracellular hemoglobin concentration and leading to HbS polymerization [13]. Considering the higher hemoglobin levels and red blood cell dehydration, HbSC disease is often described as a relative hyperviscosity phenotype [14].

Another major genotype results from the co-inheritance of HbS and β-thalassemia. In Sβ^0^-thalassemia, where the β-globin gene is completely nonfunctional, all adult hemoglobin expressed is HbS, rendering the clinical phenotype broadly comparable to homozygous HbSS disease, although distinct electrophoretic features are observed, including higher hemoglobin A_2_ and fetal hemoglobin (HbF) levels and a lower mean corpuscular volume. In Sβ^+^-thalassemia, some residual production of normal adult hemoglobin (hemoglobin A) is preserved, resulting in a generally milder phenotype. Rare β-globin variants, including HbD and HbO, may interact with hemoglobin S to produce clinically significant disease, while exceptional variants such as HbS-Antilles can cause severe sickling even in heterozygous carriers and therefore behave as autosomal dominant disorders.

Despite these well-defined genotypes, their correlation with a clinical phenotype remains imperfect. While hematologic parameters can be reasonably predicted, clinical outcomes, including the burden of acute complications, mortality, and long-term comorbidities, are influenced not only by genetic modifiers but also by social, environmental, and clinical factors. Among these, fetal hemoglobin (HbF) plays a central role in the variability and severity of the symptoms [15]. In 1948, Janet Watson hypothesized that the lack of manifestation of SCD in newborns was due to the high levels of HbF present early in life [16]. This hypothesis was later supported by observations individuals who co-inherited hereditary persistence of fetal hemoglobin (HPFH), a condition characterized by elevated HbF levels, were largely asymptomatic [17]. HbF is a strong inhibitor of HbS polymerization [18] and therefore reduces red blood cell sickling. The inhibitory effect on HbS polymerization is strongly dependent on the intracellular HbF concentration within individual erythrocytes. Many studies subsequently identified that higher levels of HbF were protective of most of SCD-related complications [19,20], including pain episodes [21], acute chest syndrome [22], stroke [23], and death [24].

Genome-wide association studies identified the transcription factor *BCL11A* as a key regulator of HbF expression and an important determinant of disease severity [25,26], establishing HbF as a major therapeutic target [25,26,27]. Hydroxyurea, an oral, daily HbF-inducing therapy has been used to modulate disease phenotype for over 30 years [28], with striking effects both HbF-dependent and independent, on clinical course, including reductions in vaso-occlusive crises (VOCs), transfusions requirements, hospitalizations, and improving survival. Additional genetic modifiers have been described, although their effects are generally modest and inconsistently demonstrated. For example, co-inheritance of α-thalassemia has been associated with a modified disease phenotype, whereas glucose-6-phosphate dehydrogenase (G6PD) deficiency has been linked in some studies to increased disease severity [15]. Several additional loci influencing HbF levels have been identified through genome-wide association studies, although their clinical significance continues to be refined [29].

As the first molecular disease, one that shaped twentieth-century medicine, SCD is now once again at the forefront of scientific and therapeutic innovation. In recent years, gene therapy has emerged as a transformative approach, opening new avenues to address the disease at its biological root. At least four broad therapeutic strategies are currently being pursued, aiming either to correct the causative mutation or to introduce hemoglobins with anti-polymerization properties capable of preventing acute and chronic manifestations. Whether these approaches constitute a true cure or rather a durable modulation of disease severity remains an open and critical question. This review will examine these strategies in detail, with particular emphasis on the underlying technologies and their clinical implications and limitations.

## 2. What Are the Possible Targets for Gene Therapy?

Multiple molecular targets have been identified for gene therapy in SCD, all aiming to reduce HbS polymerization either by increasing protective hemoglobins or by correcting the underlying β-globin mutation.

### 2.1. Fetal Hemoglobin

The beneficial effects of hydroxyurea, the first drug approved by the US Food and Drug Administration for SCD, are largely mediated through its ability to increase HbF levels [30]. Thus, increasing HbF represents a logical and promising therapeutic strategy and target for gene therapy. Such a goal appeared as potentially reachable in the early 2000 when genome-wide association studies identified single nucleotide polymorphisms (SNP) at the *BCL11A* locus as strongly associated with HbF level in general population as well as in individuals with β-thalassemia and SCD [26,31,32,33]. Additional SNP such as in the HBS1L-MYB and β-globin locus have since been identified as associated with HbF level [34], however functional studies confirmed the major role of *BCL11A*, which expression is responsible for the hemoglobin switch from fetal to adult hemoglobin [35]. Moreover, the effect of the SNPs in these three loci have very large effect, accounting for up to 50% of the HbF heritable variation [27], which is unusual for genome-wide association studies [36]. While *BCL11A* quickly appeared as a major regulator of HbF, targeting this gene required both elucidating its mechanistical effect and identifying a molecular target for gene therapy. *BCL11A* is a zinc-finger transcription factor that has been shown as a potent repressor of fetal hemoglobin [25]. Deletion of *BCL11A*, which is a cause of HPFH [37], effectively restores HbF expression [38], validating *BCL11A* loss as a potential avenue. However, as *BCL11A* has extra-erythropoietic effects including in neuron and B-cell development, erythropoietic-specific deletion is required.

**Figure 1:**
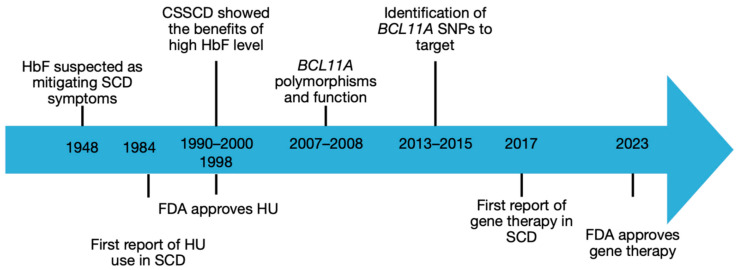
Timeline of gene therapy milestones [28,29,30,31,32,34,35,36,38,39,41,43].

One major step forward was the identification that most SNP regulating HbF level through *BCL11A* expression were located within an intronic erythroid-specific enhancer [35], offering a unique opportunity for lineage-specific deletion. Molecular dissection of this enhancer pinpointed candidate bases to target to effectively reinduce HbF expression [39]. These remarkable features supported by solid literature led to the deletion of *BCL11A* erythroid-specific enhancer as a major avenue for gene therapy in SCD and β-thalassemia (Figure 1). Of note, alternative approaches to increase HbF exist: using zinc finger nucleases [40], or short hairpin RNA as RNA interference to silence *BCL11A* [41], or targeting gamma-globin promoter [42].

### 2.2. Other Gene Therapy Targets

Since HbS sickling directly depends on HbS intracellular concentration in the red blood cells, increasing the proportion of alternative hemoglobins that do not participate in HbS polymer formation represents another potential therapeutic strategy. Several hemoglobin subtypes were investigated, including β-globin and delta-globin.

Increasing non-HbS β-globin is the aim of the FDA-approved lovotibeglogene autotemcel, the first gene therapy approach reported in 2017 [43]. This lentiviral-based approach introduces a modified β-globin gene encoding the anti-sickling hemoglobin HbA^T87Q^. This strategy leads to the production of a hemoglobin variant that reduces HbS polymerization.

Upregulating delta-globin, leading to increased HbA2 production, represents another therapeutic strategy that has shown proof of concept in mice models but remains to be tested in human [44,45].

Finally, advances in gene-editing technologies have enabled the direct correction of the causative β-globin point mutation underlying SCD in preclinical models [46,47,48].

## 3. Therapeutic Strategies and Delivery Platforms for Gene Therapy in SCD

### 3.1. Lentiviral Gene Addition Strategies

#### 3.1.1. Concept and Rationale of Lentiviral Gene Addition

Building on the therapeutic targets described above, early gene therapy strategies for SCD focused on the ectopic expression of anti-sickling globin variants in hematopoietic stem cells (HSCs). The underlying concept was that sustained expression of a functional or modified β-globin could counteract the pathogenic properties of HbS.

In this approach, autologous HSCs are genetically modified ex vivo using integrating viral vectors, enabling stable transgene expression in erythroid progeny following reinfusion and long-term hematopoietic reconstitution. Among available gene-delivery systems, lentiviral vectors rapidly emerged as the preferred approach owing to their ability to efficiently transduce quiescent HSCs while supporting durable transgene expression. In contrast to earlier γ-retroviral vectors, lentiviral systems can infect non-dividing cells and exhibit a more favorable integration profile, which contributed to their widespread adoption in HSC gene therapy [49,50].

#### 3.1.2. Engineering of Lentiviral Vectors

A major challenge in early globin gene therapy was the need to reproduce the tightly regulated expression pattern of the endogenous β-globin locus. High-level erythroid-specific expression requires regulatory elements contained within the β-globin locus control region (LCR), a powerful enhancer complex that coordinates globin gene transcription during erythroid differentiation. Incorporation of selected LCR elements into lentiviral constructs therefore represented a critical advance, allowing therapeutic levels of β-globin expression to be achieved in erythroid cells derived from transduced HSCs [50,51].

Contemporary lentiviral vectors are typically designed as self-inactivating (SIN) constructs, in which promoter activity within the long terminal repeats has been abolished, thereby reducing the risk of insertional activation of neighboring genes and improving genomic safety [52]. Additional vector optimization has focused on enhancing transduction efficiency, achieving erythroid-specific expression, and ensuring stable therapeutic globin production throughout hematopoietic differentiation.

These technological developments enabled the generation of vectors expressing modified β-globin variants with anti-polymerization properties. One of the most extensively studied constructs encodes the βA-T87Q, globin variant, which interferes with HbS polymer formation and thereby reduces erythrocyte sickling.

#### 3.1.3. Clinical Translation and Current Limitations

The clinical feasibility of lentiviral gene addition was first demonstrated in a landmark study reporting successful treatment of a patient with severe SCD using autologous HSCs transduced with a lentiviral vector encoding βA-T87Q, resulting in sustained production of therapeutic hemoglobin and marked clinical improvement [43]. This study provided proof of principle that gene therapy can achieve durable disease modification in SCD.

Despite these advances, lentiviral gene addition approaches present several intrinsic limitations. Integration of viral vectors into the host genome occurs in a semi-random manner, raising theoretical concerns regarding insertional mutagenesis, as previously observed in early γ-retroviral gene therapy trials [53]. Although lentiviral vectors exhibit a more favorable integration profile than earlier γ-retroviral vectors, the risk of insertional mutagenesis cannot be completely eliminated [52]. Indeed, cases of myelodysplastic syndrome and acute myeloid leukemia were reported during early lentiviral gene therapy trials for SCD, although subsequent analyses suggested that these events were more likely related to conditioning chemotherapy rather than vector-mediated oncogenesis. A substantial proportion of cases of myelodysplastic syndrome associated with clonal vector insertions within oncogenes and clonal evolution with acquisition of somatic genetic defects have been reported following lentiviral gene therapy for cerebral adrenoleukodystrophy, highlighting the need for continued long-term safety monitoring [54]. These events have been attributed in part to the use of a strong viral enhancer–promoter in the therapeutic vector used for CALD gene therapy, highlighting the importance of vector design in determining genotoxic risk [55].

In addition, variability in transduction efficiency and vector copy number between patients may lead to heterogeneous levels of therapeutic globin expression, potentially influencing clinical outcomes [50]. Beyond biological considerations, lentiviral gene therapy also involves substantial technical and logistical challenges, including ex vivo manipulation of autologous HSCs, large-scale vector production under good manufacturing practice conditions, and the requirement for myeloablative conditioning prior to reinfusion.

Collectively, these limitations have driven the emergence of genome editing strategies aimed at achieving more precise and physiologically regulated therapeutic outcomes.

### 3.2. Genome Editing Technologies

#### 3.2.1. Conceptual Shift: From Gene Addition to Genome Editing

More recently, advances in genome editing technologies have enabled direct targeting of the molecular pathways described above, including reactivation of fetal hemoglobin and correction of the β-globin mutation. This shift represents a transition from compensatory approaches towards potentially curative strategies that directly address the genetic basis of the disease.

Genome editing strategies aim to precisely modify disease-relevant loci, thereby enabling controlled and durable therapeutic effects [50,56]. This conceptual transition has been driven by the development of programmable nucleases capable of inducing targeted DNA cleavage at predefined genomic sites. In the context of SCD, these approaches primarily aim either to correct the pathogenic mutation in the β-globin gene or to modulate regulatory elements governing globin gene expression [56].

More recently, alternative genome editing platforms such as base editing and prime editing have been developed, enabling targeted nucleotide modifications without inducing double-strand DNA breaks [57,58]. These approaches are increasingly being explored for therapeutic applications, including in hematopoietic disorders.

#### 3.2.2. Mechanisms of CRISPR–Cas9 Genome Editing

Among genome editing platforms, CRISPR–Cas9 has emerged as the most widely adopted system owing to its programmability and efficiency. The system consists of a guide RNA (gRNA) that directs the Cas9 endonuclease to a complementary DNA sequence, where it induces a site-specific double-strand break (DSB).

Following DNA cleavage, endogenous repair pathways are engaged, primarily non-homologous end joining (NHEJ) or homology-directed repair (HDR) [59]. NHEJ is an error-prone repair mechanism that directly ligates DNA ends and frequently introduces small insertions or deletions (indels), leading to gene disruption. This property is therapeutically exploited to inactivate regulatory elements, such as enhancers controlling gene expression. In contrast, HDR enables precise sequence correction using a homologous DNA template, allowing targeted repair of disease-causing mutations. However, HDR is typically less efficient in primary hematopoietic stem cells and is largely restricted to specific phases of the cell cycle, posing challenges for therapeutic applications [48].

Importantly, the balance between NHEJ and HDR directly determines the therapeutic strategy, with NHEJ favoring robust gene disruption and HDR enabling precise gene correction.

Beyond classical CRISPR-Cas9-mediated DSB-dependent editing, base editing and prime editing systems enable targeted nucleotide modifications without inducing full double-strand breaks. Base editors use catalytically impaired Cas proteins fused to deaminases to mediate single-base conversions, whereas prime editing combines a Cas9 nickase with a reverse transcriptase to introduce precise sequence changes [57,58]. These approaches may reduce reliance on HDR and potentially improve editing outcomes in hematopoietic stem cells.

#### 3.2.3. Therapeutic Genome Editing Strategies in SCD

Genome editing enables therapeutic strategies that directly target previously defined molecular pathways in SCD, thereby allowing mechanistically informed intervention at the level of disease pathophysiology. One approach involves precise correction of the single nucleotide substitution in the β-globin gene responsible for HbS production. Proof-of-concept studies have demonstrated that CRISPR–Cas9-mediated HDR can correct the pathogenic mutation in human hematopoietic stem and progenitor cells, restoring normal β-globin expression [48].

An alternative and clinically more advanced strategy builds directly on the therapeutic rationale of fetal hemoglobin reactivation described above. Rather than targeting coding sequences, this approach exploits regulatory mechanisms governing globin gene expression. In particular, CRISPR–Cas9-mediated disruption of an erythroid-specific enhancer within the *BCL11A* locus induces robust HbF re-expression while preserving *BCL11A* function in non-erythroid lineages [39,56]. This strategy functionally recapitulates naturally occurring protective genetic variants and represents one of the most clinically advanced genome editing approaches currently under investigation in hemoglobinopathies.

Additional genome editing strategies include targeting regulatory regions within the γ-globin promoters or other loci involved in globin gene switching, although these approaches remain less advanced and are primarily evaluated in preclinical settings.

#### 3.2.4. Next-Generation Genome Editing Technologies

While CRISPR–Cas9-mediated DSBs have enabled efficient genome editing, concerns regarding genomic instability have prompted the development of alternative editing technologies. Base editing systems allow direct conversion of specific nucleotides without inducing double-strand DNA breaks, by coupling catalytically impaired Cas proteins with nucleotide deaminases. These systems enable precise nucleotide substitutions and represent a promising strategy to improve the safety profile of genome editing [57].

Prime editing represents a further refinement, combining a Cas9 nickase with a reverse transcriptase to enable targeted insertions, deletions, and base substitutions without requiring donor DNA templates or DSBs. This approach offers increased versatility and precision, although its efficiency in primary HSCs remains under investigation [58].

#### 3.2.5. Biological and Safety Challenges of Genome Editing

Importantly, despite their transformative potential, genome editing technologies raise significant safety concerns. Off-target effects, defined as unintended cleavage at genomic sites with partial sequence homology, remain a significant challenge and may lead to unintended mutations with potential oncogenic consequences.

In addition to off-target activity, CRISPR-induced DSBs can result in complex genomic alterations, including large deletions, inversions, and chromosomal rearrangements. Such events have been observed across multiple experimental systems and raise concerns regarding genomic integrity [60].

Furthermore, genome editing can activate DNA damage response pathways, including p53 signaling, which may affect cell survival and long-term engraftment of edited HSCs [61]. These findings highlight the need for continued optimization of editing strategies to improve specificity, minimize unintended genomic alterations and preserve stem cell function.

Importantly, the recently approved gene-editing therapy exagamglogene autotemcel (exa-cel) relies on CRIPSR-Cas9-mediated disruption of the *BCL11A* erythroid enhancer through NHEJ-induced indels rather than HDR-based gene correction (Frangoul et al., 2021) [62]. While this approach avoids the use of an exogenous donor DNA template and may reduce risks associated with random integration of donor sequences, it still involves the induction of DSBs and therefore does not fully eliminate the risk of genomic instability. In contrast, emerging genome editing technologies such as base editing and prime editing do not rely on full double-strand DNA breaks and may therefore mitigate some of the toxicities associated with DSB-induced genomic damage [57,58].

### 3.3. Delivery Strategies for Gene Therapy and Genome Editing

#### 3.3.1. Ex Vivo Delivery of Gene-Modified Hematopoietic Stem Cells

Current gene therapy strategies for SCD predominantly rely on ex vivo modification of autologous HSPCs, followed by reinfusion after myeloablative conditioning [48,62]. This strategy allows precise control over gene transfer or genome editing prior to transplantation.

This approach enables precise control over gene transfer or genome editing prior to transplantation and has been central to both lentiviral gene addition and CRISPR-based therapeutic platforms.

Ex vivo delivery typically involves the isolation of CD34^+^ HSPCs, followed by genetic modification using either integrating viral vectors or transient delivery of genome editing components, such as CRISPR–Cas9 ribonucleoprotein (RNP) complexes. Electroporation of RNPs has emerged as a preferred strategy, allowing efficient genome editing while limiting the duration of nuclease activity and thereby reducing off-target effects [48]. In contrast, viral delivery of genome editing machinery may result in prolonged nuclease expression, potentially increasing the risk of unintended genomic alterations.

Despite its advantages, ex vivo manipulation introduces biological constraints. Removal of HSPCs from their native bone marrow niche and exposure to in vitro culture conditions may affect cell fitness, including self-renewal capacity and long-term engraftment potential, which are critical determinants of therapeutic efficacy [50]. In addition, the requirement for myeloablative conditioning represents a major limitation, both in terms of toxicity and patient eligibility.

#### 3.3.2. Viral Delivery Systems for Genome Editing

Viral vectors remain key tools for the delivery of genome editing components or donor DNA templates, particularly in the context of homology-directed repair. Among these, adeno-associated virus (AAV) vectors are widely used due to their high transduction efficiency and relatively favorable safety profile [63].

AAV vectors are particularly well suited for delivering homologous DNA templates required for precise gene correction. However, their limited packaging capacity (approximately 4.7 kb) restricts the size of therapeutic payloads. In addition, pre-existing immunity to AAV capsids may reduce transduction efficiency and complicate their use in certain clinical settings [63].

Although AAV vectors are generally considered non-integrating, rare integration events have been reported, necessitating careful evaluation of long-term genomic safety. Importantly, emerging evidence indicates that AAV-mediated delivery of homologous repair templates may result in integration events that do not strictly follow homologous recombination mechanisms. In particular, sequences flanked by AAV inverted terminal repeats (ITRs) can be inserted at the target locus along with the ITRs themselves, rather than being precisely incorporated through HDR, leading to the introduction of exogeneous vector-derived sequences at the intended site of correction [64,65]. These atypical integration events may have unpredictable functional consequences and therefore represent an additional safety consideration.

Furthermore, when used to deliver genome editing machinery, viral systems may prolong nuclease expression, thereby increasing the potential for off-target effects. In addition, if AAV vectors are employed to deliver nucleases such as Cas9, there is a potential risk of unintended integration of Cas9-encoding sequences at the target site or elsewhere in the genome, further raising concerns regarding genomic safety [64]. These considerations have led to increasing interest in transient delivery strategies.

#### 3.3.3. Non-Viral Delivery Platforms

To overcome the limitations associated with viral vectors, substantial efforts have been directed towards the development of non-viral delivery systems. These include lipid nanoparticles (LNPs), polymer-based carriers, and physical delivery methods such as electroporation, which enable intracellular delivery of nucleic acids or protein complexes without genomic integration [66].

LNP-based systems have gained considerable attention due to their clinical success in other therapeutic areas and their ability to deliver RNA or RNP complexes in a transient and controllable manner. Preclinical studies have demonstrated that LNP-mediated delivery of CRISPR components can achieve efficient gene editing in vivo, although translation to hematopoietic stem cells remains challenging [67].

Non-viral delivery platforms offer several advantages, including reduced immunogenicity and greater flexibility in design. However, achieving efficient and specific targeting HSCs, particularly within the bone marrow niche, remains a major obstacle. In addition, optimization of intracellular trafficking and editing efficiency continues to be an active area of investigation.

#### 3.3.4. In Vivo Delivery: Emerging Perspectives and Challenges

In vivo delivery of gene therapy and genome editing tools represents a major objective for the field, with the potential to bypass ex vivo manipulation and conditioning regimens. Such approaches could simplify therapeutic workflows and broaden the applicability of gene-based treatments.

However, in vivo delivery faces substantial biological and technical challenges. Efficient targeting of rare HSC populations within the bone marrow microenvironment remains difficult, and systemic delivery raises concerns regarding off-target editing in non-hematopoietic tissues. In addition, immune responses directed against delivery vectors or genome editing components may limit efficacy and durability [63].

As a result, although in vivo strategies are conceptually attractive, they remain largely at the preclinical stage, and significant technological advances will be required to enable their clinical translation.

#### 3.3.5. Delivery as a Central Bottleneck in Gene Therapy

Taken together, delivery represents a central determinant of the safety and efficacy of gene therapy and genome editing approaches in SCD. While ex vivo strategies currently provide the most robust and clinically validated framework, they are associated with important biological and technical constraints.

Viral and non-viral delivery systems each offer distinct advantages and limitations, and their optimization remains a key focus of ongoing research. Importantly, the complexity of current delivery strategies has significant implications for the broader implementation of gene therapy, as will be discussed in subsequent sections. Thus, continued innovation in delivery technologies will be essential to fully realize the therapeutic potential of gene therapy for SCD.

## 4. Existing Clinical Data on Gene Therapy in SCD

Clinical evidence supporting gene therapy in SCD has emerged primarily from early-phase clinical trials and subsequent multicenter studies, most commonly designed as phase I/II open-label, single-arm interventional trials. More recently, phase II/III pivotal studies have provided confirmatory efficacy data and contributed to regulatory approvals in some jurisdictions. These studies are summarized in Table 1.

### 4.1. Lentiviral Gene Addition

Clinical results from lentiviral gene therapy trials in SCD have demonstrated sustained expression of therapeutic hemoglobin and encouraging clinical efficacy signals. The most extensive clinical data derive from studies of LentiGlobin-based therapy developed by bluebird bio, evaluated in trials such as NCT02140554 and NCT04293185 and discussed in detail below.

#### 4.1.1. Study Design, Populations, and Number of Participants

As discussed above, the first clinical proof-of-concept for lentiviral gene therapy in SCD was reported in 2017 with treatment of a patient using the LentiGlobin BB305 vector encoding the modified β-globin variant HbAT87Q [43].Subsequent studies expanded this approach in multicenter phase I/II trials evaluating lovotibeglogene autotemcel (lovo-cel), a lentiviral gene therapy product based on the same vector system [68]. Across these trials, 47 patients with severe SCD have been treated. Participants enrolled in these studies were adolescents and adults (12–50 years) with severe disease manifestations, including recurrent vaso-occlusive crises (VOCs), acute chest syndrome, or other severe complications despite standard therapy. Initial protocols excluded patients with severe cerebrovascular disease, including overt stroke, significant intracranial vasculopathy, moyamoya disease, or abnormal transcranial Doppler velocities. However, eligibility criteria evolved over time, and later cohorts included patients with prior overt stroke; in the pivotal HGB-206 Group C cohort, 5 treated patients had a history of overt stroke [68].

#### 4.1.2. Clinical Outcomes

Clinical efficacy has been evaluated using both hematologic endpoints and clinical outcomes. Hematologic parameters include total hemoglobin levels, expression of therapeutic hemoglobin, and markers of hemolysis. Clinical outcomes have focused primarily on frequency of vaso-occlusive crises, episodes of acute chest syndrome, transfusion requirements, and hospitalization rates.

Across studies, most treated patients experienced substantial reductions in vaso-occlusive events, and many achieved complete elimination of severe crises during follow-up. Improvements in hemoglobin concentration and reductions in hemolysis markers have also been consistently reported.

#### 4.1.3. Duration of Follow-Up

Follow-up durations vary across trials but now extend to more than five years in some patients treated in early-phase studies, demonstrating sustained expression of therapeutic hemoglobin and durable clinical benefit.

#### 4.1.4. Other Lentivirus-Based Gene-Therapy Programs

The γ-globin-expressing ARU-1801 vector developed by Aruvant Sciences has also been evaluated in a first-in-human phase 1/2 study (NCT02186418) using reduced-intensity conditioning prior to autologous transplantation of lentivirally modified HSC. The vector encodes a modified γ-globin gene producing the anti-sickling fetal hemoglobin HbF^G16D. In seven treated patients with 2–7 years of follow-up, sustained expression of HbF^G16D was observed and was associated with greater than 80% reduction in severe vaso-occlusive events, with a short period of severe cytopenias (median grade 4, neutropenia: 8 days; thrombocytopenia: 5 days). Although the study met its predefined feasibility and safety endpoints, it was terminated after treatment of seven patients once these objectives were achieved, and larger studies are needed to further evaluate the potential advantages of reduced-intensity conditioning [69].

The BCH-BB694 approach developed at Boston Children’s Hospital targets erythroid *BCL11A* to induce endogenous HbF production through a lentiviral vector expressing a microRNA-adapted short hairpin RNA. In a first-in-human clinical study, autologous CD34^+^ hematopoietic stem progenitor cells (HSPC) were transduced ex vivo and reinfused following myeloablative conditioning. Treated patients exhibited robust HbF induction (approximately 20–40%) with a high proportion of F-cells and marked clinical improvement, including substantial reductions in vaso-occlusive events during follow-up [41]. These results provided proof-of-concept that erythroid-specific repression of *BCL11A* can therapeutically reactivate HbF in patients with SCD. Ongoing studies are further evaluating this strategy and its long-term safety (NCT05353647).

Other programs, including the DREPAGLOBE study conducted by Assistance Publique–Hôpitaux de Paris using the GLOBE1 vector (NCT03964792) [70] and the UCLA study evaluating the Lenti/G-βAS3-FB vector (NCT02247843) [71], remain in earlier stages of clinical evaluation with limited publicly available efficacy data.

Overall, these trials provide proof-of-concept that lentiviral gene addition or HbF reactivation can achieve meaningful clinical benefit in severe SCD, although longer follow-up is needed to fully assess durability and long-term safety.

### 4.2. CRISPR–Cas9/12 Genome Editing

Genome editing using CRISPR–Cas9 has emerged as a second major gene therapy strategy for SCD, aiming to modify endogenous genetic elements that regulate hemoglobin expression rather than introducing an exogenous globin gene. The most clinically advanced CRISPR-based approach targets the erythroid-specific enhancer of *BCL11A*, a transcription factor that represses HbF) expression after birth. Disruption of this enhancer in hematopoietic stem cells leads to sustained reactivation of endogenous HbF production, which inhibits hemoglobin S polymerization and reduces red blood cell sickling.

Reni-cel (EDIT-301) is an investigational autologous gene-editing therapy that uses CRISPR-Cas12a to reactivate fetal hemoglobin through targeted editing of the HBG1 and HBG2 promoters, thereby mimicking naturally occurring hereditary persistence of fetal hemoglobin (HPFH) variants. Unlike CRISPR-Cas9 approaches targeting the *BCL11A* enhancer, reni-cel directly edits the γ-globin promoters to restore endogenous HbF expression [72].

#### 4.2.1. Study Design, Populations, and Number of Participants

Clinical evaluation of CRISPR-based genome editing has primarily involved autologous hematopoietic stem cells edited ex vivo using CRISPR–Cas9 ribonucleoprotein complexes targeting the erythroid-specific *BCL11A* enhancer. The edited cells are reinfused following myeloablative conditioning with busulfan.

The most extensively studied CRISPR-based therapy is exagamglogene autotemcel (exa-cel), formerly known as CTX001, developed through a collaboration between Vertex Pharmaceuticals and CRISPR Therapeutics. This therapy has been evaluated in multicenter clinical trials including NCT03745287 and NCT03655678 [73].

Participants enrolled in these studies were adolescents and adults with severe SCD characterized by recurrent vaso-occlusive crises despite standard therapy. Eligibility criteria were broadly similar to those used in lentiviral gene therapy trials, generally excluding individuals with severe end-organ damage or advanced cerebrovascular disease. Across these trials, several dozen patients have now undergone CRISPR-mediated gene editing and autologous stem cell transplantation.

#### 4.2.2. Clinical Outcomes

Clinical results reported to date demonstrate robust induction of fetal hemoglobin following genome editing of the *BCL11A* enhancer. Treated patients typically achieve HbF levels exceeding 30–40%, with a large proportion of erythrocytes expressing HbF (F-cells). This HbF induction is associated with marked reductions in hemolysis markers and substantial increases in total hemoglobin concentration.

Importantly, most treated patients have experienced complete elimination of severe vaso-occlusive crises during follow-up, accompanied by significant reductions in hospitalization and transfusion requirements. These findings provide strong clinical evidence that targeted disruption of the *BCL11A* erythroid enhancer can effectively reactivate endogenous HbF and ameliorate the clinical manifestations of SCD.

In ongoing clinical studies with Reni-cel, 28 patients with severe sickle cell disease have been treated to date. Reported outcomes demonstrated robust and durable HbF induction, with mean HbF levels approaching 48%, associated with marked reductions in hemolysis and near-complete elimination of vaso-occlusive events in treated cohorts. Early data also demonstrated successful engraftment and favorable hematologic recovery following myeloablative conditioning. Reni-cel remains in active clinical development, and longer-term follow-up is required to determine durability of response, long-term safety, and effects on chronic organ complications [72,74].

#### 4.2.3. Duration of Follow-Up

Follow-up durations in CRISPR-based gene editing trials currently extend to several years in some participants. Available data indicates durable HbF expression and sustained clinical benefit without evidence of loss of edited hematopoietic stem cell engraftment. Continued long-term monitoring remains essential to assess durability of therapeutic effects and potential late adverse events associated with genome editing.

### 4.3. Base Editing

Risto-cel (BEAM-101) is an investigational ex vivo gene-editing therapy using adenine base editing of the HBG1 and HBG2 promoters to inhibit *BCL11A* binding. In the ongoing BEACON phase I/II study, 31 patients with severe sickle cell disease have been treated with autologous hematopoietic stem cells edited using an adenine base editor. Early clinical results demonstrated robust and durable fetal hemoglobin induction, with HbF levels frequently exceeding 60% and marked suppression of HbS production. These biologic effects were associated with rapid hematologic improvement and, to date, no reported severe vaso-occlusive events following engraftment. Longer follow-up remains necessary to evaluate durability, long-term safety, and effects on chronic organ complications [75].

**Table 1:** Gene and genome-editing clinical trials for sickle cell disease by therapeutic strategy.

Therapeutic Strategy	Product/Candidate	Molecular Target/Mechanism	Platform/Vector	Clinical Experience	Key Findings	Current Status	Reference
Lentiviral Gene Addition (anti-sickling β-globin)	Lyfgenia (lovo-cel)	Addition of modified β-globin (HbAT87Q)	Lentiviral BB305 vector	>80–100 treated worldwide; 47 infused in pivotal dataset	Durable therapeutic Hb production; major reduction or elimination of severe VOEs; long-term follow-up up to 60 months	FDA approved (2023)	Ribeil et al., 2017 [43] Kanter et al., 2022 [68] Kanter et al., 2023 [76]
	Lenti/G-βAS3-FB	Addition of anti-sickling β-globin variant (βAS3)	Lentiviral vector	4 treated	Clinical improvement in most treated patients; some achieved transfusion independence	Development discontinued	Prueksaproapong et al., 2026 [71]
Lentiviral HbF Reactivation	ARU-1801	γ-globin addition (HbFG16D)	Lentiviral vector + reduced-intensity conditioning	7 treated	Sustained HbF expression with >80% reduction in severe VOEs and shorter cytopenias	Phase 1/2 completed	Grimley et al., 2025 [69]
	BCH-BB694	Erythroid-specific *BCL11A* silencing	Lentiviral shRNA/miRNA-adapted vector	6 patients with published follow-up	HbF induction ~20–40%; marked reduction in VOCs	Active clinical development	Esrick et al., 2021 [41]
	DREPAGLOBE (GLOBE1 vector)	γ-globin expression	Lentiviral GLOBE1 vector	4 patients with published follow-up	Variable clinical benefit; partial loss of corrected cells upon engraftmen; 2 patients transfusion-independent	Ongoing early clinical evaluation	Sobrino et al. 2025 [70]
CRISPR-Cas9 *BCL11A* Editing (HbF Reactivation)	Casgevy (exa-cel)	Disruption of erythroid-specific *BCL11A* enhancer	CRISPR-Cas9 ex vivo editing	44 treated in major studies; pediatric expansion ongoing	Robust HbF induction (>30–40%); 97% patients free of VOEs	Approved in US/UK/EU/Canada	Frangoul et al., 2024 [73]
	BIVV003 (SAR445136)	*BCL11A* enhancer disruption	Zinc Finger Nucleases (ZFN)	6 treated	Increased HbF and reduction in disease manifestations	Program terminated	Lessard et al., 2024 [40]
	OTQ923	*BCL11A* editing	CRISPR-Cas9	3 treated	Early HbF induction and reduction in VOEs	Development discontinued	Sharma et al. 2023 [77]
CRISPR-Cas12 Editing (HbF Reactivation)	Reni-cel (EDIT-301)	*HBG1*/*HBG2* promoter editing to mimic HPFH variants	CRISPR-Cas12a	28 treated	Mean HbF ~48%; near-complete elimination of VOEs in reported cohort	Ongoing clinical development	Hanna et al., 2023 [74] Hanna et al., 2024 [72]
Base Editing Technologies	Risto-cel (BEAM-101)	Mimics hereditary persistence of fetal hemoglobin (HPFH) variants	Adenine base editor	31 treated	HbF frequently >60%; suppression of HbS; no severe VOEs reported post-engraftment	Active BEACON study	Gupta et al., 2026 [75]
	CS-101	HbF induction	Transformer base editor (tBE)	1 reported patient	Rapid engraftment and durable HbF induction	Recruiting in China	ClinicalTrials.gov ID NCT07000318 [78]
Direct β-globin Gene Correction	Nula-cel (GPH101)	Direct correction of HbS mutation	CRISPR-Cas9 + AAV6 donor template	1 treated	Initial proof-of-concept with marked HbF induction and VOE resolution	Program paused/restructured	Kanter et al., 2021 [79]

Abbreviations: HbF = fetal hemoglobin; HbS = sickle hemoglobin; HPFH = hereditary persistence of fetal hemoglobin; VOC = vaso-occlusive crisis.

## 5. SCD Gene Therapy: Who and How?

Both exa-cel and lovo-cel carry broad indications: any patient aged 12 years or older with SCD and a history of recurrent VOCs [62]. In practice, selection criteria applied by experienced centers are considerably more restrictive, as stated in recent publications including in the joint European Hematology Association (EHA) and European Bone Marrow Transplantation (EBMT) Hemoglobinopathies Working Party consensus statement published in 2025. Identifying patients who are candidates for gene therapy requires medical, logistical, financial, and psychological determinants [77,80].

### 5.1. Medical Candidate Selection for Gene Therapy

With the current approved indication, patients who are candidates for gene therapy generally present with severe SCD (HbSS, HbS/B^0^, HbS/B^+^, or severe HbSC phenotype) with documented history of at least two severe VOCs per year in the preceding 24 months, or with prior stroke, recurrent acute chest syndrome, or progressive end-organ damage. They must have adequate cardiopulmonary, hepatic, and renal function to tolerate myeloablative conditioning, no active uncontrolled infections, and either no HLA-matched sibling donor or a documented preference for an autologous approach. The EHA-EBMT panel recommends offering gene therapy before irreversible organ impairment has progressed to a point that excludes safe conditioning.

Several clinical situations represent relative or absolute contraindications. Patients with advanced and irreversible end-organ damage, including severe pulmonary arterial hypertension, dilated cardiomyopathy, hepatic cirrhosis (often from transfusional iron overload), or severe chronic kidney disease (stage 4 or 5) face high risk from high-dose chemotherapy. However, this applies particularly to adults with SCD who frequently accumulate subclinical organ damage over decades. A history of hematological malignancy or myelodysplastic syndrome is a relative contraindication, in particular for lentiviral vector-based products [81]. Poor HSPC mobilization, which is common after prolonged hydroxyurea use, can compromise product yield and manufacturing feasibility [77]. Patients with established severe cerebrovascular disease, including moyamoya angiopathy, were generally excluded from pivotal trials, as it is not clear whether gene therapy can adequately address pre-existing neurological injury [81]. Pregnancy and breastfeeding are absolute contraindications, and children under 12 years are currently off-label. The fitness criteria and contraindications for autologous gene therapy in SCD patients are summarized in Table 2.

In practice, attrition between initial referral and product infusion is high. Pre-existing organ dysfunction, failed mobilization, logistical complexity of the 8 to 12-month pathway, and patient withdrawal all contribute to dropout rates that are substantially higher than trial enrolment figures suggest [77,82].

### 5.2. Leukapheresis and Myeloablative Conditioning

Mobilized autologous HSPCs are collected by apheresis. A minimum CD34^+^ cell yield of at least 2 to 3 × 10^6^ cells/kg is required for product manufacturing, with an additional unmodified aliquot preserved as back-up in case of engraftment failure if enough cells are available. This threshold is frequently difficult to achieve in SCD patients, as the disease itself disrupts the bone marrow niche and prolonged prior hydroxyurea use further impairs stem cell mobilization, representing one of the main causes of attrition between patient selection and product infusion [77,81]. Several medications must be discontinued before HSPC mobilization, specifically hydroxyurea and crizanlizumab at least 2 months before leukapheresis, and iron chelators 3 to 6 months before infusion. Granulocyte colony-stimulating factor (G-CSF) is contraindicated for mobilization in SCD because of the risk of life-threatening sickling events; therefore, plerixafor is used instead, typically over multiple apheresis cycles to achieve sufficient CD34^+^ cell yield.

Gene therapy requires myeloablative conditioning regimen. Reduced-intensity conditioning regimens have been explored to extend eligibility to patients with existing end-organ damage, but at the cost of lower vector copy numbers and potentially lower therapeutic hemoglobin levels [77]. The current high-dose regimen consists of intravenous busulfan (cumulative dose of approximately 12.8 mg/kg) administered every 6 h over 4 consecutive days (days −6 to −3), followed by a 48 to 72-h washout period before infusion of the gene therapy product. Pharmacokinetic monitoring after the first dose of busulfan allows dose adjustment to target optimal drug exposure according to CASGEVY Full Prescribing Information. Busulfan-based myeloablative conditioning has been used extensively for allogenic hematopoietic stem cell transplant (HSCT) and is known to be associated with acute toxicities including severe mucositis, febrile neutropenia, and prolonged thrombocytopenia [83], as well as a risk of hepatic veno-occlusive disease that is heightened in SCD patients (children more than adults) with pre-existing iron overload or liver damage [84,85]. Seizure prophylaxis is required during administration given the epileptogenic properties of busulfan, and gonadal toxicity leading to infertility makes fertility preservation before conditioning a standard component of pre-treatment care.

### 5.3. A Patient-Centered Decision-Making

The current management of SCD includes disease-modifying therapies, chronic transfusion programs, allogeneic HSCT, including reduced-intensity conditioning regimen, and now autologous gene therapy. Choosing among these options requires individualized reasoning that accounts for disease severity, organ function, donor availability, and patient values and preferences.

#### Allogenic HSCT vs. Gene Therapy in SCD

For patients with a mild-to-moderate phenotype who respond adequately to hydroxyurea with meaningful VOC reduction and acceptable HbF levels, the risk-benefit ratio of all-HSCT or myeloablative gene therapy is difficult to justify. For patients with inadequate response, are intolerant, or cannot sustain adherence, alternative therapy is a reasonable option.

In pediatric patients with SCD who receive matched-sibling allogeneic HSCT, event-free survival and overall survival exceed 90%, with follow-up data spanning more than 30 years [84,85,86,87]. In children with a well-matched sibling donor and severe disease, allogenic HSCT is generally considered the reference curative option given the length of follow-up and the established risk profile. For adults, transplant-related morbidity is higher, and less than 20% of patients have a matched sibling donor. Haploidentical HSCT has expanded access, yet carries higher rates of graft-versus-host disease (GvHD) and is associated with worse outcomes than matched-sibling transplantation, although post-transplant cyclophosphamide has improved outcomes similar to matched-sibling allogenic HSCT [88]. Autologous gene therapy avoids GvHD and removes the need for donor identification. Reduced-intensity conditioning regimens combining low-dose total body irradiation (TBI, 300 cGy) with alemtuzumab (Campath) have expanded access to allogeneic HSCT in both children and adults by reducing transplant-related toxicity and allowing mixed chimerism, which is sufficient for disease control in SCD. However, this approach carries a risk of graft rejection and requires prolonged immunosuppression, and cases of myelodysplastic syndrome and acute leukemia have been reported after non-myeloablative allogeneic HSCT in SCD patients in both age groups, a finding that complicates direct safety comparisons with autologous gene therapy [89,90,91].

Whether gene therapy will eventually replace allogenic HSCT as first-line curative therapy, or remain primarily for patients without suitable donors, will depend on long-term follow-up data and global accessibility. Updated lovo-cel data show overall and event-free survival rates comparable to those reported for matched-sibling HSCT [82,92,93], but this comparison requires prospective validation. We propose a clinical decision frame for therapy selection in SCD in Table 3 that accounts for clinical and ethical factors.

### 5.4. Psychosocial, Socioeconomic, and Accessibility Challenges

The clinical results summarized above are not sufficient to determine who can benefit from gene therapy and must be read alongside the socioeconomic and structural constraints affecting patients with SCD.

#### 5.4.1. Health System and Financial Barriers

The global distribution of SCD is a critical determinant to consider. Approximately 300,000 infants are born with SCD each year worldwide, with more than 5 million affected individuals living in sub-Saharan Africa and approximately 1 million in India [94], while access to gene therapy is currently limited to a small number of centers in the North-America and Europe. The specialized manufacturing infrastructure, cold chain logistics, and skilled workforce required for ex vivo autologous cell therapies are not available in low- and middle-income countries [94,95]. Intellectual property structures governing CRISPR technologies may further restrict access [96], in a pattern similar to one that delayed HIV treatment in low-income countries [95]. In vivo delivery might overcome most problematics related to ex vivo manipulation and allow better global access but might also not be available across all centers and still requires a very specific medical expertise [97].

Approved gene therapy infusion products cost approximately 2 to 3 million USD per bag of genetically modified cells [98]. These amounts do not include the total cost of care, which includes multiple hospitalizations, apheresis procedures, conditioning, management of post-infusion aplasia, and more than a decade of mandatory follow-up.

Even in high-income countries, access to gene therapy remains uncertain. In the United States, the Centers for Medicare and Medicaid Services launched an outcomes-based reimbursement model in January 2025, but coverage for Medicaid patients varies by state and private insurers have added eligibility criteria more restrictive than the FDA labels. In Canada, Health Canada approved exa-cel in September 2024 and Canada’s Drug Agency issued a conditional reimbursement recommendation in January 2025, but final coverage decisions rest with each province and none had confirmed funded access as of March 2026. In France, early access authorization was granted in 2024, then withdrawn at the manufacturer’s request following a disagreement over the health technology assessment outcomes and no definitive reimbursement decision has been reached. These examples illustrate that regulatory approval does not translate automatically into patient access, even in countries with universal health coverage.

#### 5.4.2. Psychological Considerations

SCD predominantly affects populations of African descent, groups with a documented history of marginalization within biomedical research and healthcare systems [94,99]. These inequities are directly relevant to questions of access, informed consent, and the distribution of benefits from gene therapy. The gene therapy pathway is demanding from a psychosocial standpoint with 8 to 12 months from initial evaluation to product infusion, multiple hospitalizations and a prolonged period of aplasia and uncertainty. For some patients and families, SCD is not only a disease but a central part of social identity and community belonging. Research in the HSCT context has documented a form of identity disruption following curative therapy, and similar experiences have been reported after gene therapy [100,101]. Stigma, particularly in healthcare environments where Black patients are underrepresented, may also affect access to referrals, participation in clinical trials, and quality of follow-up.

### 5.5. Research and Clinical Perspectives

#### 5.5.1. Current Pitfalls and Need for Trials

The approval of exa-cel and lovo-cel in 2023 marked a turning point in academic output and in this field. The number of publications addressing gene therapy for SCD has increased considerably, with a high volume of clinical trial reports, health technology assessments, and equity analyses in the past 3 years. Several research priorities remain inadequately addressed. Long-term follow-up data beyond 5 years are available for only a minority of treated patients, and the durability of gene correction over a patient’s lifetime has not been established [62,73]. Furthermore, the risk for secondary neoplasia, including myelodysplasia and leukemia, remains to be assessed in the long term. Comparative effectiveness studies between autologous gene therapy and allogeneic HSCT with reduced-intensity conditioning are needed, with current comparisons being indirect and based on non-contemporaneous cohorts. The effect of gene therapy on established organ damage, including nephropathy, retinopathy, and cerebrovascular disease, has not been studied as a primary endpoint in any published trial.

#### 5.5.2. The Patient’s Perspective

Patient-reported outcomes represent another gap. Current quality of life data rely on instruments that were not designed specifically for the post-gene therapy context and do not capture identity disruption, psychosocial adaptation, or the long-term lived experience of patients who have undergone a potentially curative procedure for a disease that shaped their social world [100,101,102]. Validated and longitudinal approaches integrating patient narratives alongside quantitative endpoints are needed.

#### 5.5.3. Financial and Ethical Responsibilities

From a health systems research standpoint, the economics of gene therapy in SCD are poorly characterized outside the United States. Budget impact analyses for other countries with universal health coverage are limited, as are studies on the real-world implementation capacity of specialized centers, the logistical determinants of patient attrition, and the cost of the full treatment pathway including post-infusion follow-up. In low- and middle-income countries, where the disease burden is greatest, the research infrastructure to generate local evidence is nearly absent, creating a self-reinforcing gap between disease prevalence and scientific production [94,95,97,103].

A multi-disciplinary expert group has published target product profiles (TPP) defining the minimal and optimal requirements that ex vivo and in vivo gene therapies for SCD should meet to be considered clinically and globally viable. TPP covers meaningful aspects of sustained, efficient and responsible gene therapy for SCD, i.e., a clinical efficacy threshold, an acceptable safety profile including risks associated with conditioning, accessibility in low-resource settings, and acceptability from the patient perspective including treatment burden and quality of life [104]. These profiles are intended to guide the design of next-generation clinical trials and to ensure that future products address not only the clinical needs of patients in high-income countries but also the constraints of the settings where most affected individuals live.

#### 5.5.4. Access to Gene Therapy in Younger Children with SCD

Pediatric data in patients under 12 years are limited but promising. Recently, preliminary data from the Phase 3 CLIMB SCD-151 study in 10 children aged 5 to 11 years that received exa-cel to treat SCD showed that all 4 evaluable patients achieved outcomes similar to data from adults and adolescents [105]. Global regulatory submissions for this age group are expected in the first half of 2026. No pediatric extension trial for children below 12 years has been initiated for lovo-cel.

### 5.6. Future Perspectives and Development

Despite major progress, gene therapy for SCD remains in an early phase of clinical implementation. Several scientific, clinical, and structural challenges must be addressed to ensure that these approaches translate into durable and widely accessible treatments.

From a scientific standpoint, continued innovation in genome engineering technologies is expected to refine both the safety and precision of therapeutic strategies. Emerging platforms such as base editing and prime editing may enable targeted nucleotide correction without introducing double-strand DNA breaks, thereby reducing the risk of genomic instability. In parallel, advances in delivery technologies and improvements in hematopoietic stem cell editing efficiency will be essential to achieve stable engraftment and durable therapeutic hemoglobin expression.

Clinically, long-term follow-up remains critical. Current data extend beyond five years for only a minority of treated patients, and lifetime monitoring will be necessary to assess the durability of gene correction and the risk of late adverse events such as clonal hematopoiesis or secondary malignancies. Another key priority is the reduction in treatment-related toxicity. Current protocols rely on myeloablative conditioning with agents such as busulfan, which significantly contributes to treatment morbidity and restricts eligibility for many patients. The development of non-genotoxic or antibody-based conditioning strategies could substantially expand the safety profile of gene therapy. In addition, future studies should assess the effect of gene therapy on established organ complications and include pediatric populations, particularly younger children in whom early intervention may prevent irreversible disease manifestations.

Beyond the clinical setting, the implementation of gene therapy raises broader health system and equity considerations. The current ex vivo autologous model requires specialized infrastructure, complex manufacturing processes, and prolonged clinical monitoring, limiting access to a small number of centers worldwide. Given that the global burden of SCD is concentrated in low- and middle-income countries, improving accessibility will be a central challenge for the coming decade. Simplified manufacturing pathways, in vivo delivery approaches, and international collaborations will be critical to ensure that gene therapy evolves not only as a technological achievement but also as an equitable therapeutic option.

## 6. Conclusions

**Figure 2:**
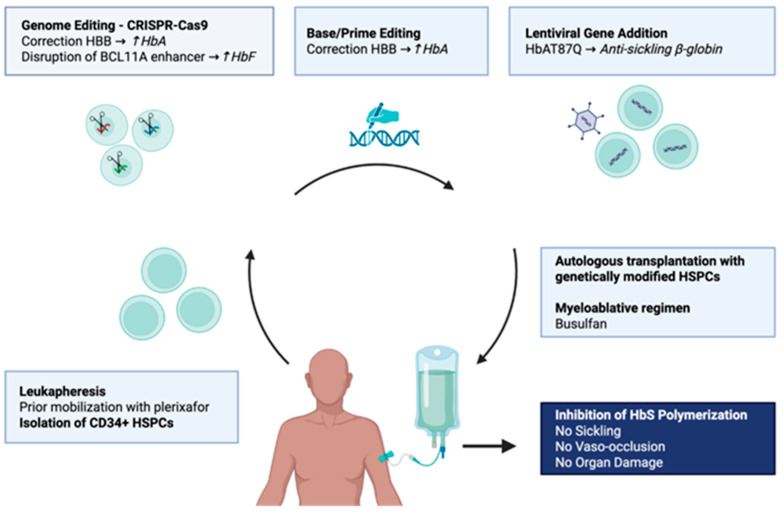
Overview of gene therapy approaches in SCD. Hb: hemoglobin; HSPC: hematopoietic stem progenitor cell. Created in BioRender. Tardif, M. (2026) https://BioRender.com/z6fpk7o (accessed on 15 May 2026).

Gene therapy has transformed the therapeutic landscape of SCD by providing a potentially curative autologous treatment capable of eliminating vaso-occlusive crises in most treated patients (Figure 2). However, clinical efficacy alone does not determine its place in patient care. Treatment decisions remain highly individualized and must consider disease severity, organ function, donor availability, and patient preferences.

Major challenges remain, including long-term safety monitoring, comparative effectiveness with allogeneic transplantation or between different gene therapy approaches, and the evaluation of outcomes beyond acute complications, particularly quality of life and chronic organ damage. Moreover, the complexity and cost of current gene therapies limit their accessibility to the SCD population.

Future advances in genome editing technologies, non-genotoxic conditioning, and simplified delivery strategies may help address these limitations. Ultimately, the success of gene therapy in SCD will depend not only on continued scientific innovation but also on the development of equitable strategies that allow these therapies to reach the populations most affected by the disease.

## Figures and Tables

**Table 2:** Eligibility criteria and contraindications for autologous gene therapy in SCD [1,6,7,8,11].

Criterion	Indication	Contraindication
Disease severity	At least 2 severe VOCs per year; stroke; recurrent ACS; progressive organ damage	Mild-moderate phenotype well-controlled on hydroxyurea
Age	12 years or older (approved indication)	Under 12 years (off-label; trials ongoing)
Organ function	Adequate hepatic, cardiac, pulmonary, renal functions	Advanced PAH, cardiomyopathy, cirrhosis, CKD stage 4–5
Donor availability	No HLA-matched sibling donor	Available matched sibling (allo-HSCT may be preferred)
Hematological history	No prior hematological malignancy or MDS	Prior AML or MDS; alpha-thalassemia trait (lovo-cel only)
HSPC mobilization	Adequate CD34^+^ yield	Poor mobilization; ongoing contraindicated medications
CNS	No severely established cerebrovascular disease	Moyamoya angiopathy; severe stroke sequelae
Reproductive status	Not pregnant; fertility preservation completed or discussed	Pregnancy; breastfeeding

ACS: acute chest syndrome; AML: acute myeloid leukemia; CKD: chronic kidney disease; CNS: central nervous system; HSPC: hematopoietic stem and progenitor cells; MDS: myelodysplastic syndrome; PAH: pulmonary arterial hypertension; VOC: vaso-occlusive crisis.

**Table 3:** Proposed clinical decision structure for curative therapy selection in SCD.

Clinical Context	«Preferred» Approach	Alternative	Rationale
Child under 12 years, matched sibling, severe SCD	Allogenic HSCT	Gene therapy trial if available	Over 30 years of follow-up; matched sibling reduces GvHD
Adult 12 years or older, severe SCD, no matched donor	Gene therapy	Haploidentical HSCT	Autologous; no GvHD risk; no donor required
Any age, severe SCD, matched sibling available	Shared decision (gene therapy vs allogenic HSCT)	Optimize disease-modifying therapy	Patient preference; center experience; age-related risk
Moderate SCD, hydroxyurea-responsive	Optimize hydroxyurea	Consider adding crizanlizumab	Avoid myeloablation toxicity in non-severe phenotype
Severe SCD with significant organ damage	Disease-modifying therapy and supportive care	Gene therapy or HSCT if organ function allows	Myeloablation risk assessed individually
Low- or middle-income country, severe SCD	Hydroxyurea; transfusions	Allogenic HSCT if available	Gene therapies currently inaccessible (see Section 5.4)

## Data Availability

No new data were created or analyzed in this study. Data sharing is not applicable.

## Abbreviations

The following abbreviations are used in this manuscript:

SCD	Sickle cell disease Digital Publishing Institute
HbS	Hemoglobin S
HbF	Fetal hemoglobin
HPFH	hereditary persistence of fetal hemoglobin
VOC	Vaso-occlusive crises
G6PD	Glucose-6-phosphate dehydrogenase
HSC	Hematopoietic stem cells
LCR	Locus control region
NHEJ	Non-homologous end joining
HDR	Homology-directed repair
